# *In vitro* toxicological characterisation of arsenic-containing fatty acids and three of their metabolites†
†Electronic supplementary information (ESI) available. See DOI: 10.1039/c5tx00122f



**DOI:** 10.1039/c5tx00122f

**Published:** 2015-07-03

**Authors:** S. Meyer, G. Raber, F. Ebert, L. Leffers, S. M. Müller, M. S. Taleshi, K. A. Francesconi, T. Schwerdtle

**Affiliations:** a Graduate School of Chemistry , University of Münster , Wilhelm-Klemm-Straße 10 , 48149 Münster , Germany . Email: tanja.schwerdtle@uni-potsdam.de; b Institute of Nutritional Science , University of Potsdam , Arthur-Scheunert-Allee 114-116 , 14558 Nuthetal , Germany; c Institute of Chemistry – Analytical Chemistry , NAWI Graz , University of Graz , Universitätsplatz 1 , 8010 Graz , Austria; d Heinrich-Stockmeyer-Stiftung , Parkstraße 44-46 , 49214 Bad Rothenfelde , Germany; e Department of Marine Chemistry , Faculty of Marine Science , University of Mazandaran , Babolsar , Iran

## Abstract

Arsenic-containing fatty acids are a group of fat-soluble arsenic species (arsenolipids) which are present in marine fish and other seafood. Recently, it has been shown that arsenic-containing hydrocarbons, another group of arsenolipids, exert toxicity in similar concentrations comparable to arsenite although the toxic modes of action differ. Hence, a risk assessment of arsenolipids is urgently needed. In this study the cellular toxicity of a saturated (AsFA 362) and an unsaturated (AsFA 388) arsenic-containing fatty acid and three of their proposed metabolites (DMA^V^, DMAPr and thio-DMAPr) were investigated in human liver cells (HepG2). Even though both arsenic-containing fatty acids were less toxic as compared to arsenic-containing hydrocarbons and arsenite, significant effects were observable at μM concentrations. DMA^V^ causes effects in a similar concentration range and it could be seen that it is metabolised to its highly toxic thio analogue thio-DMA^V^ in HepG2 cells. Nevertheless, DMAPr and thio-DMAPr did not exert any cytotoxicity. In summary, our data indicate that risks to human health related to the presence of arsenic-containing fatty acids in marine food cannot be excluded. This stresses the need for a full *in vitro* and *in vivo* toxicological characterisation of these arsenolipids.

## Abstract 2

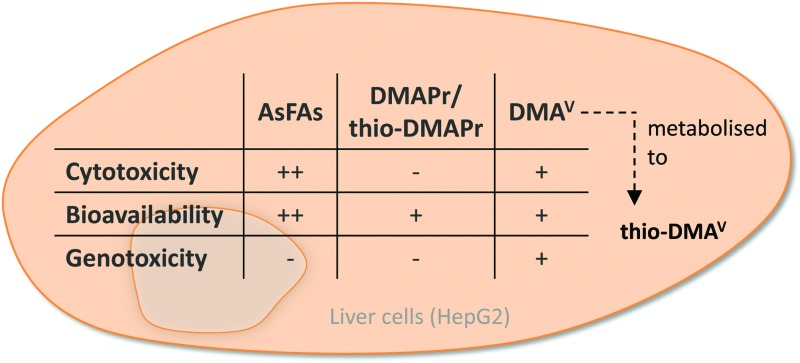
Arsenic-containing fatty acids are bioavailable and toxic to human liver cells in culture.

## Introduction

Diet is the primary source of arsenic intake in the general population. In marine food the arsenic content is up to 100-fold higher (1–100 mg kg^–1^) than in terrestrial food and arsenic is mostly present as organic species. Apart from arsenobetaine and arsenosugars around 10–50% of the total arsenic can occur as arsenolipids.^1,2^

In the last two decades a variety of lipid-soluble arsenic species have been identified and their structures have been confirmed. The classes of arsenolipids include arsenic-containing fatty acids (AsFAs),^3^ arsenic-containing hydrocarbons (AsHCs),^4^ arsenosugar-phospholipids (AsPLs)^5^ as well as cationic trimethylarsonio fatty alcohols (TMAsFOHs).^6^ Very recently conjugated compounds thought to be wax esters or more likely glycerides were reported in the less polar fraction of an extract from blue whiting oil.^7^

AsFAs consist of a polar dimethylarsinoyl group and a carboxylic acid with a long hydrocarbon chain in the middle, which can be saturated or unsaturated. AsFAs were identified in several cod liver oil samples,^3,8^ in the liver of northeast arctic cod,^9^ in edible fish like herring^10^ or red mullet^11^ and some brown algae.^12^

Whereas inorganic arsenic (iAs) is classified as a human carcinogen (group 1) by the International Agency for Research on Cancer (IARC) and lots of data exist about its toxic and health related effects,^13^ less is known about the effects of arsenolipids.^1^ Recently, it has been shown that three AsHCs exerted toxic effects on human urothelial and liver cells in similar concentrations compared to effects caused by arsenite (iAs^III^). However, toxic modes of action seem to be different.^14^ The same three AsHCs have also been investigated in the *in vivo* model organism *Drosophila melanogaster*. In contrast to iAs^III^ the AsHCs had an impact on the late development stages of the fruit fly, especially by preventing the hatching of flies of the F1 generation out of the pupae.^15^ Toxicological data for the other three groups of arsenolipids are not yet available, although the European Food Safety Authority (EFSA) had already concluded in 2009 that a risk assessment of arsenolipids in seafood is urgently needed.^16^

Arsenolipids are bioavailable to humans and are thoroughly biotransformed. After consumption of arsenolipid-containing cod liver oil, dimethylarsinic acid (DMA^V^) was identified as the main metabolite (up to 70%) in the urine of two volunteers.^17,18^ This arsenical is also the major metabolite of iAs^III^ ^19^ and is classified as possibly carcinogenic to humans (group 2B) by the IARC.^13^ It exerts genotoxicity in cultured human cells^20,21^ and induces bladder cancer in rats.^19^ Further urine metabolites of arsenolipids are oxo- and thio-derivatives of dimethylarsenobutanoic acid (DMAB, thio-DMAB) and dimethylarsenopropanoic acid (DMAPr, thio-DMAPr).^17,18^

Having a look at the biosynthesis route of arsenolipids DMAPr is probably one of the major substrates when AsFAs are formed by marine organisms, *e.g.* algae. The AsFAs are lengthened by two carbon units from acetyl coenzyme A following the elongation of non-arsenic-containing fatty acids during their biosynthesis.^2,3^ The same substrate unspecificity can be responsible for the shortening of AsFAs to DMAPr and DMAB during beta-oxidation in human fatty acid catabolism.

**Fig. 1:**
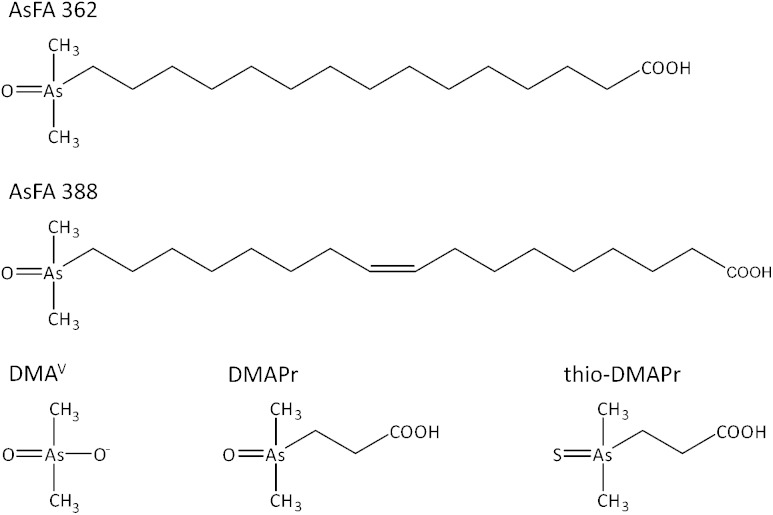
Chemical structures and abbreviations of arsenic species investigated in this study.

In this study the cytotoxicity, bioavailability and genotoxicity of a saturated (AsFA 362) and an unsaturated arsenic-containing fatty acid (AsFA 388) (Fig. 1) were investigated for the first time in human liver cells (HepG2). In addition, the toxicity of the three metabolites DMA^V^, DMAPr and thio-DMAPr (Fig. 1) was characterised in human liver cells and urothelial (UROtsa) cells.

## Experimental

### Materials

Minimal essential medium Eagle (MEM), non-essential amino acids (NEA) and culture dishes were provided by Biochrom (Berlin, Germany). Fetal calf serum (FCS) was purchased from PAA Laboratories (Pasching, Austria). Penicillin–streptomycin solutions, trypsin, cacodylic acid (DMA^V^, ≥99% purity) and hydrogen peroxide solution (30%, Suprapur) were products of Sigma Aldrich (Steinheim, Germany). Nitric acid (65% Suprapur) was from Merck (Darmstadt, Germany). Sodium(*meta*)-arsenite (≥99% purity) and Alcian Blue were from Fluka Biochemika (Buchs, Germany). The cell-counting kit-8 (CCK-8) was obtained from Dojindo molecular technologies (Munich, Germany) and the inductively coupled plasma mass spectrometry (ICP-MS) elemental standard (As, 1 mg L^–1^) from Spetec (Erding, Germany). All other chemicals were of p.a. grade and were from Merck (Darmstadt, Germany) or Sigma Aldrich (Steinheim, Germany). HepG2 liver cells were supplied by the European Collection of Cell Cultures (ECACC; number 85011430, Salisbury, UK). The urothelial cell line UROtsa was derived from a primary culture of a normal human urothelium through immortalisation with the SV-40 large T antigen. This cell line was kindly provided by Prof. M. Stýblo (University of North Carolina, USA).

### Synthesis and preparation of arsenic-containing fatty acids and their metabolites for cytotoxicity studies

The arsenic-containing fatty acids were synthesised and purified as reported previously.^22^ The purity of the compounds was >99% determined by NMR spectroscopy and HPLC coupled with ESI-MS and ICP-MS.

### Cell culture and incubation with arsenicals

HepG2 and UROtsa cells were grown in culture dishes as a monolayer using MEM containing FCS (10%, v/v), penicillin (100 U mL^–1^) and streptomycin (100 μg mL^–1^). For HepG2 the medium was additionally supplemented with non-essential amino acids (NEA) (1%, v/v). Cultures were incubated at 37 °C with 5% CO_2_ in the air with 100% humidity. Cells were seeded in a defined density (17 000 cells per cm^2^) for each experiment and were incubated with the respective arsenical for 48 h after 24 h of logarithmic growing.

Stock solutions (10 mM) of the three metabolites were prepared in bi-distilled water and stock solutions of the arsenic-containing fatty acids were prepared in 100% EtOH. They were stored at 4 °C. Shortly before each experiment the stock solutions were diluted with bi-distilled water or EtOH, respectively. In experiments where EtOH was used as the solvent, the EtOH concentration was set to 1%. No cytotoxic effects compared to untreated control cells were seen at this concentration (data not shown). Notably, the incubation of cells with 1% EtOH resulted in an increase of total cellular arsenic. This is likely to result from slight disturbance of cellular membrane integrity by the solvent and thus an increased uptake of arsenic, which is present in the culture media at a concentration of 0.39 μg As L^–1^.

### Cytotoxicity testing

The cytotoxicity of the arsenic-containing fatty acids and their metabolites was elucidated by quantifying their effects on cell number, lysosomal integrity as well as dehydrogenase activity.

Effects on cell number were studied after 48 h of incubation as reported before^23^ by the use of an automatic cell counter (Casy TTC®, Roche Innovatis AG). Results were calculated as percentage of control.

Viable cells are able to incorporate and bind the supravital dye neutral red in their lysosomes.^24^ Therefore after incubation the medium was replaced by a neutral red (3-amino-7-dimethylamino-2-methylphenazine hydrochloride) containing medium (55.6 mg L^–1^ neutral red in MEM). After 3 h of dye loading the incorporated dye was solubilised in 100 μL of acidified EtOH solution (50% EtOH, 1% acetic acid in PBS) and the absorbance was measured using a plate reader (Tecan Infinite M200® PRO, Tecan, Germany) at 540 nm.

Dehydrogenase activity as an additional cell viability marker was assessed colorimetrically applying the cell-counting kit-8 (CCK-8) as described previously.^25^ In brief, after 48 h of incubation with the respective compound HepG2 cells were incubated for 1 h with WST-8 solution (2-(2-methoxy-4-nitrophenyl)-3-(4-nitrophenyl)-5-(2,4-disulfo-phenyl)-2*H*-tetrazolium). Absorbance was determined at 450 nm using a plate reader (Tecan Infinite M200® PRO, Tecan, Germany).

### Cellular bioavailability and distribution of arsenic

Cellular bioavailability and distribution were studied as described before.^14^ Briefly, after 48 h of incubation trypsinised cells were pelletised and for total arsenic measurement wet-ashed (acid digestion with HNO_3_/H_2_O_2_ solution (1/1, v/v) at 95 °C for at least 12 h). The arsenic content of the digest was determined by ICP-MS/MS (Agilent 8800 ICP-QQQ, Agilent Technologies, Germany) in the mass-shift mode using oxygen as the reaction gas. ICP-MS/MS parameters are listed in Table 1. According to the calculated LOQ a cellular arsenic concentration of at least 0.08 μM is quantifiable using the described method when 380 000 cells are seeded.

**Table 1:** ICP-MS/MS parameters

Forward power	1550 W
Cool gas flow	15 L min^–1^
Auxiliary gas flow	0.9 L min^–1^
Nebulizer gas flow	1 L min^–1^
Nebulizer type	MicroMist
Quadrupole 1	*m*/*z* 75
Reaction gas flow	O_2_: 0.3 mL min^–1^ (purity 99.9999%)
Quadrupole 2	*m*/*z* 91
Integration time	1 s
LOD^*a*^	3.0 ng L^–1^
LOQ^*a*^	12.0 ng L^–1^

^*a*^DIN 32645.

For distribution analysis, cell pellets were lysed by addition of bi-distilled water and sonication (15 s, 100%, 0.8 cycles). The cytosol was separated from the cell-debris-associated parts by centrifugation (5 min, 23 600*g*, 4 °C) and the total arsenic content of both fractions was determined by ICP-MS/MS as described above.

### Analysis of water-soluble arsenic species

Analysis of arsenic species was carried out as previously reported by LC-ICP-MS/MS.^26^ Briefly, incubated and pelletised cells were resuspended in bi-distilled water and dissolved by ultrasonication (15 s, 100%, 0.8 cycles). After centrifugation (5 min, 23 600*g*, 4 °C) the supernatant was injected into the LC-ICP-MS/MS system (Agilent Technologies, Germany) for separation and quantification of arsenic species. Determination was performed at 40 °C with a Hamilton PRP-X100 column (4 × 150 mm, particle size 10 μm). The mobile phase was 20 mM ammonium carbonate/formic acid buffer (pH 8) (flow rate: 1 mL min^–1^, injection volume: 20 μL). ICP-MS/MS was used in the mass shift mode.

For confirmation of arsenic species exact mass was determined by LC-ESI-HRMS (Q-Exactive, Thermo Scientific, Germany). Separation was carried out at 30 °C with an Atlantis dC18 column (4.1 × 150 mm, particle size 5 μm) (Waters Corporation, USA) with 20 mmol formic acid (pH 3) as the mobile phase (flow rate: 1.0 mL min^–1^, injection volume: 20 μL). The ESI-MS was equipped with an atmospheric pressure ionisation source employing electrospray nebulisation with nitrogen as the nebuliser gas. Measurements were performed in positive mode, with a drying gas temperature of 350 °C, a spray voltage of 3.2 kV and a resolution chosen at 70 000. The mass range was set to *m*/*z* = 137–141 (DMA^V^) and *m*/*z* 153–157 (thio-DMA^V^) without additional fragmentation.

Since our early experiments showed that arsenic species are not stable during storage of cellular extracts, in all studies LC-ICP-MS/MS analysis was always carried out immediately after the lysis of cells to avoid artefacts.^27,28^

### Genotoxicity testing – micronuclei formation

Micronuclei formation was investigated as described before.^29^ In brief, cells were seeded on Alcian blue-coated glass coverslips and incubated with the respective arsenical for 48 h, fixed with an ice-cold fixation solution (90% MeOH, 10% PBS, –20 °C) and stained with acridine orange (125 mg L^–1^ in PBS). Micronuclei formation was evaluated by fluorescence microscopy. As earlier studies indicate that several arsenicals interact with the formation of the spindle apparatus or the effect of cytochalasin B, the application of cytochalasin B was dropped.^20^ Cell proliferation was monitored by cell number quantification and to ensure mitosis an incubation time of 48 h was chosen, which is in accordance with around 2 cell cycles of untreated control cells.

### Statistics

All experiments were carried out at least three times, each time on a different day. As indicated in the respective figure captions, from the raw data the mean standard deviation (SD) was calculated and a statistical analysis was performed by using the ANOVA-one way-test. Significance levels are *p < 0.05, **p < 0.01 and ***p < 0.001.

## Results

### Cytotoxicity

**Fig. 2:**
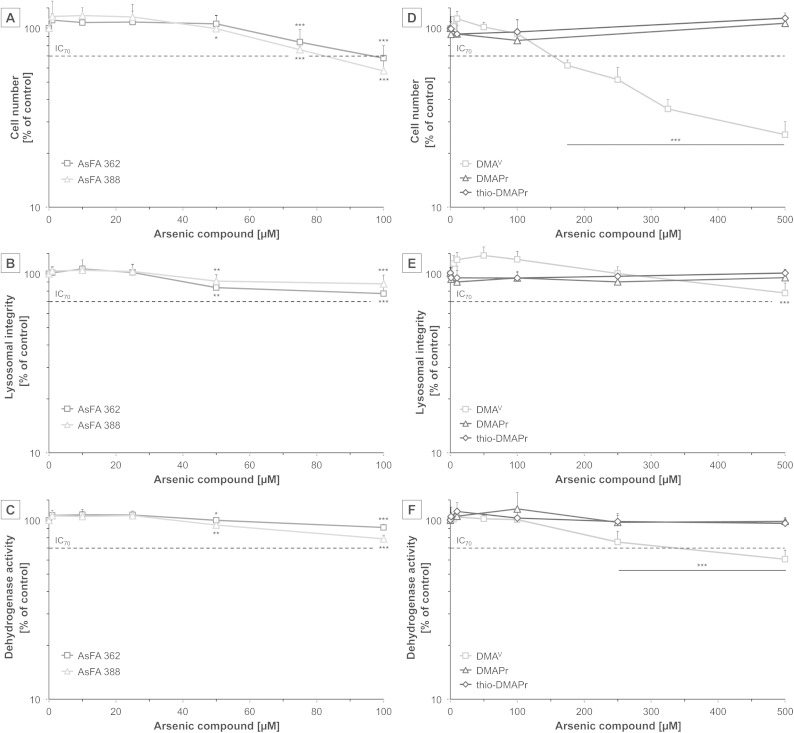
Cytotoxicity of two arsenic-containing fatty acids (AsFA 362 and AsFA 388 (A–C)) and three of their metabolites (DMA^V^, DMAPr and thio-DMAPr (D–F)) in HepG2 cells after 48 h of incubation. In the case of AsFAs data were normalised to solvent control, 1% EtOH (100%), which exerted no cytotoxicity itself. Cytotoxicity was determined by the impact on cell number (A and D), lysosomal integrity as measured by neutral red uptake (B and E) and dehydrogenase activity as measured by the CCK-8 assay (C and F). Shown are mean values of at least three independent determinations +SD. *p < 0.05, **p < 0.01, ***p < 0.001.

To assess cytotoxicity, effects of the respective arsenic compounds on cell number, lysosomal integrity and dehydrogenase activity were examined after 48 h of incubation in human liver cells (HepG2). In the case of arsenic-containing fatty acids (AsFAs), cell number was identified as the most sensitive endpoint (Fig. 2A–C). For all tested vitality endpoints significant effects were observable at 50 μM and higher. Compared to the toxic arsenical reference arsenite, effects caused by the AsFAs were in a 10-fold higher concentration range than effects caused by arsenite (Table 2).

**Table 2:** IC_70_ values of the investigated cytotoxicity endpoints after 48 h of incubation with two AsFAs, their three tested metabolites and iAs^III^ for comparison in HepG2 cells. The IC_70_ represents the inhibitory concentration of compounds that are required for 30% reduction of the respective cytotoxicity marker

	Cell number	Lysosomal integrity	Dehydrogenase activity
AsFA 362	96 μM	>100 μM	>100 μM
AsFA 388	83 μM	>100 μM	>100 μM
DMA^V^	155 μM	>500 μM	335 μM
DMAPr	>500 μM	>500 μM	>500 μM
thio-DMAPr	>500 μM	>500 μM	>500 μM
iAs^III^	9 μM	25 μM	25 μM

Next, the cytotoxic effects of the three metabolites DMA^V^, DMAPr and thio-DMAPr were assessed (Fig. 2D–F). Interestingly, DMAPr and thio-DMAPr did not cause any significant effects up to 500 μM. DMA^V^ affected the endpoint cell number with an IC_70_ of 155 μM, thereby exerting slightly lower cytotoxicity than the AsFAs. Both vitality marker lysosomal integrity and dehydrogenase activity were less sensitive.

Because all three applied metabolites have been identified in the urine of humans^17,18^ their cytotoxicity was additionally investigated in a human urothelial (UROtsa) cell line. In former experiments these cells exerted higher sensitivity towards both arsenic-containing hydrocarbons and arsenite as compared to HepG2 cells. Here, DMAPr and thio-DMAPr exerted no significant cytotoxic effects in UROtsa cells up to an incubation of 500 μM (ESI – Fig. S1†). However, cytotoxicity was observable for DMA^V^, as reported before with IC_70_ values of 205 μM, 249 μM and 319 μM for the endpoints cell number, dehydrogenase activity and lysosomal integrity, respectively.^29^

### Cellular bioavailability and distribution

The cellular arsenic concentrations were determined after 48 h of incubation with the arsenicals in HepG2 cells. Both AsFAs were strongly bioavailable (Table 3). After incubation with 1 μM of AsFA 362 or AsFA 388, the cellular arsenic concentration was around 20-fold higher as compared to the incubation concentration. In the case of incubation with 100 μM of the respective AsFAs, the cellular concentration was even around 80-fold higher as compared to the incubation concentration. Thereby, 58% (AsFA 362) and 53% (AsFA 388) of the total arsenic were identified as arsenic in the cytosolic fraction (Table 4). This is in strong contrast to the water soluble DMA^V^, where about 82% of the total arsenic was located in the cytosolic fraction.

**Table 3:** Cellular concentration of arsenic in HepG2 cells after 48 h of incubation with the respective arsenic species [μM]

Incubation [μM]	0	1	10	25	50	100
AsFA 362	2.3 ± 1.2^*a*^	17 ± 1	157 ± 18	610 ± 80	1909 ± 191	7552 ± 310
AsFA 388	2.3 ± 1.2^*a*^	21 ± 5	149 ± 23	599 ± 77	2208 ± 176	8726 ± 1582
DMA^V^	0.3 ± 0.1	—	11.3 ± 3.0	—	46 ± 12	73 ± 19
DMAPr	0.3 ± 0.1	0.3 ± 0.2	0.3 ± 0.1	—	—	1.6 ± 0.1
Thio-DMAPr	0.3 ± 0.1	0.4 ± 0.1	1.2 ± 0.3	—	—	16 ± 6

^*a*^Solvent controls: cells were incubated with 1% ethanol.

**Table 4:** Cellular distribution of arsenic after 48 h incubation with 50 μM of the respective arsenic compounds in HepG2 cells

	Cell-debris associated fraction	Cytosol-fraction	Total	% of total As^*a*^
AsFA 362:	41.8 ± 0.9%	58.2 ± 0.9%	1999 ± 43.1 μM	104.7%
AsFA 388:	46.7 ± 2.6%	53.3 ± 2.6%	1627 ± 40.5 μM	73.7%
DMA^V^:	17.8 ± 3.1%	82.2 ± 3.1%	54 ± 3.6 μM	117.4%

^*a*^Cellular bioavailability.

The metabolites DMA^V^, DMAPr and thio-DMAPr were much less bioavailable than the AsFAs (Table 3). Remarkably, cellular arsenic concentrations were 5- to 10-fold higher after incubation with thio-DMAPr than with its oxo analogue DMAPr. This thio-related effect has already been shown for DMA^V^ and thio-DMA^V^ in UROtsa cells.^29^ However, cellular arsenic concentrations after incubation with DMAPr and thio-DMAPr were much lower than the incubation concentration. DMA^V^ was more bioavailable in HepG2 cells than DMAPr and thio-DMAPr, with cellular arsenic concentration being about equimolar in relation to the incubation concentration.

**Fig. 3:**
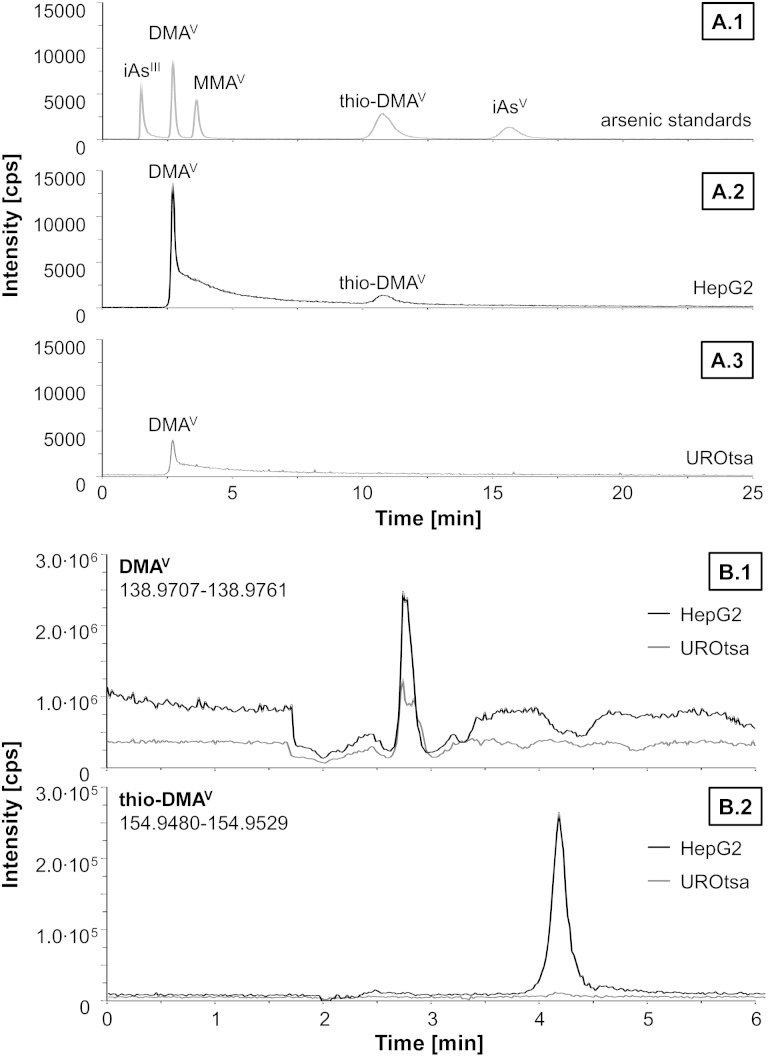
Representative chromatograms of separation and identification of arsenic species by LC-ICP-MS/MS (A) and LC-ESI-HRMS (B) after incubation of HepG2 and UROtsa cells with 50 μM DMA^V^ for 48 h. A1: separation of a mixture of arsenic standards containing arsenite (iAs^III^), dimethylarsinic acid (DMA^V^), monomethylarsonic acid (MMA^V^), thio-dimethylarsinic acid (thio-DMA^V^) and arsenate (iAs^V^) in concentration of 0.1 μM. A2: arsenic species in the soluble part of lysat from HepG2 cells. A3: arsenic species in the soluble part of lysat from UROtsa cells. B1: DMA^V^ trace of cell lysat. B2: thio-DMA^V^ trace of cell lysat.

Since DMA^V^ exerted cytotoxic effects on HepG2 cells in similar concentrations as compared to UROtsa cells,^29^ which are in general more sensitive towards arsenicals, speciation analysis was carried out on both cell lines after incubation with DMA^V^ to get an idea about cellular DMA^V^ metabolism (Fig. 3). HepG2 cells metabolised DMA^V^ partly to thio-DMA^V^, which has been classified as highly cytotoxic in former studies.^20,29,30^ Interestingly, thio-DMA^V^ was not observed as a DMA^V^ metabolite in UROtsa cells. Measurements were carried out by LC-ICP-MS/MS and thio-DMA^V^ was identified by exact mass analysis determined by LC-ESI-HRMS. Determined masses of DMA^V^ and thio-DMA^V^ were in good correlation with their calculated masses and both compounds demonstrated identical retention times in samples and standards in LC-ICP-MS/MS and LC-ESI-HRMS experiments.

### Genotoxicity

**Fig. 4:**
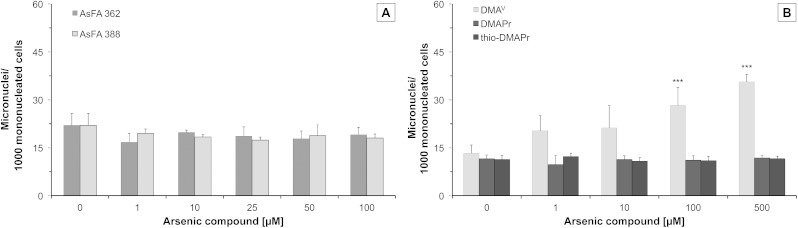
Formation of micronuclei in HepG2 cells after 48 h of incubation with two arsenic-containing fatty acids (AsFA 362 and AsFA 388 (A)) and three of their metabolites (DMA^V^, DMAPr and thio-DMAPr (B)). Displayed are mean values of at least three independent determinations +SD. ***p < 0.001.

To further toxicologically characterise the arsenic species, we studied micronuclei formation, as a marker for genotoxicity at the chromosomal level. For the two As-FAs, thio-DMAPr and DMAPr, no significant micronuclei induction was observed. Only DMA^V^ was able to promote the formation of micronuclei after 48 h of incubation (Fig. 4).

## Discussion

In this study, the cellular toxicity and bioavailability of two food-relevant arsenic-containing fatty acids (AsFAs), which represent one group of arsenolipids, and three water-soluble metabolites of arsenolipids were tested for the first time. A human liver cell line (HepG2) was chosen as an *in vitro* model because the liver is proposed as the site of arsenolipid metabolism.

Thereby, the saturated AsFA 362 as well as the unsaturated AsFA 388 caused significant cytotoxicity. However, these two fatty acids were around 10 to 20-fold less toxic than the AsHCs tested with the same cell line.^14^ For AsHCs it has been proposed, that they can interact with the membranes of a cell, because of their amphiphilic structure consisting of a polar dimethylarsinoyl head group and a lipophilic hydrocarbon tail. Consequently, their structure is comparable to that of fatty acids, which are components of membrane lipids.^14^ In contrast, AsFAs have two polar head groups, the dimethylarsinoyl group and the carboxylic acid group, resulting in a lower structural accordance to membrane lipids. This fact might explain their lower cellular bioavailability and in consequence the lower cytotoxicity as compared to AsHCs. When the cells were incubated with AsFAs, arsenic accumulated especially in the cell-debris-associated fraction of a cell, a result similar to that found for AsHCs. The cellular total arsenic concentrations following AsFA incubation, however, were around 2.5 to 5-fold lower than after incubation with equal concentrations of AsHCs.^14^ As these two types of arsenolipids probably undergo passive transport, involving either simple or facilitated diffusion, their structural similarity to, and interaction with, membrane lipids might play a major role in their uptake and resulting toxicity. Thus, less similarity caused by the two polar groups in the structure of AsFAs results in a lower accumulation and cytotoxicity, because the interaction with cell membranes is lower.

However, recently it has been proposed that AsFAs are also conjugated to wax esters or to triglycerides.^7^ These compounds have a higher lipophilic character, which supports their cellular uptake. Consequently, after intracellular hydrolysis of these compounds the cellular concentrations can be higher promoting the cytotoxic effects of AsFAs. This enhanced transport has to be taken into account in the final risk assessment of AsFAs.

Neither of the two AsFAs exerted genotoxic effects at the chromosomal level as determined by micronuclei formation. In addition, no increase of bi- or multinucleated cells occurred (data not shown), indicating that in the observed concentration range the arsenicals do not cause a mitotic arrest, which might have explained their cytotoxicity. This is in contrast to arsenite, which has been previously shown, in the same cell line and also other cellular systems, to cause micronuclei formation as well as an increased number of bi- and multinucleated cells in the low cytotoxic concentration range.^14,31,32^

After two volunteers consumed cod liver oil, which naturally contains a mixture of arsenolipids, they excreted in their urine DMA^V^, DMAPr, and thio-DMAPr.^17,18^ In addition, DMAPr occurs in a variety of marine samples and it has been proposed as an intermediate product in the biosynthetic route of arsenobetaine and other organic arsenic species.^33^

Whereas DMAPr and thio-DMAPr did not exert any cytotoxic or genotoxic effects at exposures up to 500 μM (the concentration of these compounds in urine after cod liver oil consumption was only 0.01–0.03 μM^17^) the main metabolite DMA^V^ had a significant impact, especially on the cell number. These DMA^V^ induced effects occurred in a similar, but slightly higher concentration range, as compared to the applied AsFAs. Regarding the endpoint cytotoxicity, the metabolism of both AsHCs and AsFAs to DMA^V^, DMAPr and thio-DMAPr might be categorised as a detoxification process. Nevertheless, it has to be taken into account that in contrast to AsFAs, their major metabolite DMA^V^ caused significant genotoxicity in this study, as well as in several previous studies.^20,21^ Moreover, DMA^V^ is known to cause bladder cancer in rats.^19^ Therefore, from a chronic toxicity point of view, we cannot exclude that metabolism of AsFAs is a toxification process.

The cytotoxicity order of the three metabolites can be ascribed to their bioavailability. In HepG2 cells the cellular arsenic concentration after incubation with DMA^V^ was up to 10-folds higher than after incubation with thio-DMAPr, which was in turn more bioavailable than its oxo analogue DMAPr. Whereas the lipophilic AsFAs probably get into the cell by passive diffusion, the water-soluble arsenicals need a transport system. For DMA^V^ and other pentavalent arsenicals like thio-DMA^V^ aquaglyceroporins are discussed as transporters.^34^ Arsenite is also a substrate of this integral membrane protein channel.^35^ In contrast to the three metabolites, iAs^III^ is able to accumulate in HepG2 cells by a factor of 6–7.^14^ Different uptake rates can be explained by different p*K*_a_ values of a molecule and as a result its dissociation state. Therefore the charge of a molecule is very important for this transportation process and uncharged molecules can cross the cell membrane *via* membrane channels much faster.^34,36^ Whereas iAs^III^ is uncharged at physiological pH, DMA^V^ is partly dissociated, which explains the differences in the uptake rates of these two compounds.^37^ Although DMAPr and thio-DMAPr are both pentavalent arsenicals, they probably do not have a specific transporter, and hence their uptake rates are low. Interestingly, the thio analogue is a factor 10 more bioavailable than the oxo form. This polarity-related accumulation was also observed for other oxo and thio arsenicals like DMA^V^ and thio-DMA^V^ ^31^ or DMA^V^–sugar–glycerol and DMA^V^–sugar–sulphate.^29,38^

In this study however, we show that DMA^V^ is metabolised in HepG2 cells to its thio analogue thio-DMA^V^. This is an explanation for the higher cytotoxicity observable in this cell line compared to UROtsa cells, especially since thio-DMA^V^ has been demonstrated before to exert massive cellular toxicity.^19,27,28^

A similar transformation to dimethyldithioarsinic acid (dithio-DMA^V^) has been observed *in vitro* when DMA^V^ was incubated in a liver homogenate. It was proposed in this study that DMA^V^ is first reduced to DMA^III^ and dithio-DMA^V^ is then formed by reaction with sulfane sulphur, because dithio-DMA^V^ was mainly found after incubation with DMA^III^.^39^ However, thio-DMA^V^ is also found in the urine of humans and animals exposed to arsenic; its *in vitro* toxicity is comparable to that shown by trivalent arsenic species and is much higher than that of other pentavalent oxo arsenicals.^19,27,28^ The high toxicity might be caused by production of reactive oxygen species through the redox equilibrium between DMA^V^ and DMA^III^.^28,40^ The observed cytotoxic effects of DMA^V^, especially after 48 h of incubation, might be caused by thio-DMA^V^ – found as a metabolite in HepG2 cells, but not in UROtsa cells – or the proposed intermediate DMA^III^ formed by the metabolism of DMA^V^ to its thio analogue. This might be an explanation for the high cytotoxicity of DMA^V^ observed in HepG2 cells compared to UROtsa cells, which are in general more sensitive.

## Conclusions

Saturated and unsaturated AsFAs exert cytotoxicity in human liver cells, although they are less toxic than iAs^III^ and do not show any genotoxic effects. The main metabolite of arsenolipids, DMA^V^, caused effects in a slightly higher concentration range than AsFAs, probably because it is metabolised to thio-DMA^V^, its highly toxic thio analogue. However, two other metabolites DMAPr and thio-DMAPr were less cytotoxic, which can be ascribed to their low bioavailability.

In contrast to other organic arsenic species like arsenobetaine and arsenosugars, it could be shown that AsFAs as well as AsHCs as previously reported^14,15^ have a toxic potential. Consequently, a risk to the human health by arsenolipids cannot be excluded and further experiments, for example in experimental animals, are necessary to complete the toxicological data set for a final risk assessment of arsenolipids.

## Conflict of interest

The authors declare no conflict of interest.
