# Supplementary material for: In vitro toxicological characterisation of arsenic-containing fatty acids and three of their metabolites
Source: Toxicol Res (Camb). 2015 Jul 3;4(5):1289–96. doi: 10.1039/c5tx00122f (PMC4690163; doi:10.1039/c5tx00122f)
Supplement: Supplementary file 1 [file TX-004-C5TX00122F-s001.pdf]

Electronic Supplementary Material (ESI) for Toxicology Research.
This journal is © The Royal Society of Chemistry 2015

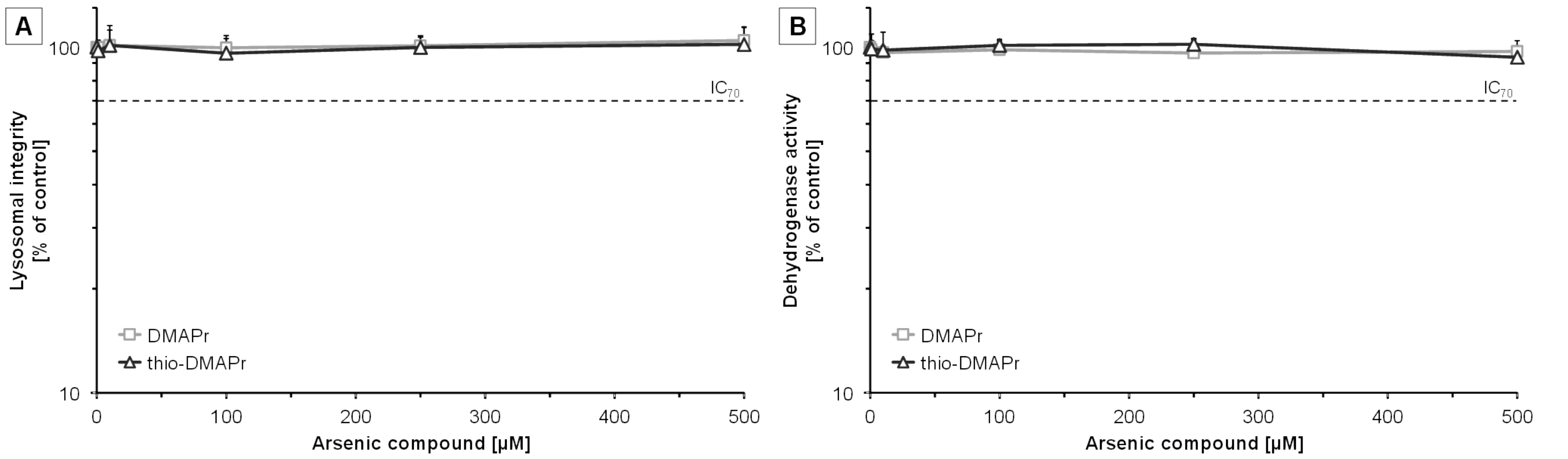

Figure S1. Cytotoxicity of DMAPr and thio-DMAPr in UROtsa cells after 48 h of incubation. Cytotoxicity was determined by impact on lysosomal integrity as measured by neutral red uptake (A) and dehydrogenase activity as measured by the CCK-8 assay (B). Shown are mean values of at least three independent determinations +SD normalised to untreated control cells.
